# Do polyproline II helix associations modulate biomolecular condensates?

**DOI:** 10.1002/2211-5463.13163

**Published:** 2021-05-02

**Authors:** Miguel Mompeán, Javier Oroz, Douglas V. Laurents

**Affiliations:** ^1^ Departamento de Química Física Biológica Instituto de Química Física Rocasolano CSIC Madrid España

**Keywords:** biomolecular condensates, polyproline II helix, SH3 domain

## Abstract

Biomolecular condensates are microdroplets that form inside cells and serve to selectively concentrate proteins, RNAs and other molecules for a variety of physiological functions, but can contribute to cancer, neurodegenerative diseases and viral infections. The formation of these condensates is driven by weak, transient interactions between molecules. These weak associations can operate at the level of whole protein domains, elements of secondary structure or even moieties composed of just a few atoms. Different types of condensates do not generally combine to form larger microdroplets, suggesting that each uses a distinct class of attractive interactions. Here, we address whether polyproline II (PPII) helices mediate condensate formation. By combining with PPII‐binding elements such as GYF, WW, profilin, SH3 or OCRE domains, PPII helices help form lipid rafts, nuclear speckles, P‐body‐like neuronal granules, enhancer complexes and other condensates. The number of PPII helical tracts or tandem PPII‐binding domains can strongly influence condensate stability. Many PPII helices have a low content of proline residues, which hinders their identification. Recently, we characterized the NMR spectral properties of a Gly‐rich, Pro‐poor protein composed of six PPII helices. Based on those results, we predicted that many Gly‐rich segments may form PPII helices and interact with PPII‐binding domains. This prediction is being tested and could join the palette of verified interactions contributing to biomolecular condensate formation.

## Graphical abstract

Polyproline II helices and the domains that bind them are common in biology. Often connected in tandem, their associations can drive the formation of biomacromolecular condensates. Glycine‐rich polyproline II helices can combine to form polyproline II helical bundles and might then associate with drive phase transitions.

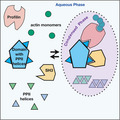

**Abbreviations**
CPEB: cytoplasmic polyadenylation element‐binding (protein)
CTD: C‐terminal domain
PRM: proline‐rich motif
RNA pol II: RNA polymerase II
sfAFP: snow flea antifreeze protein
SH3: Src‐homology 3 (domain)
VASP: vasodilator‐stimulated phosphoprotein
WASP: Wiskott–Aldrich syndrome protein
WW: tryptophan–tryptophan (domain)
ZnF: zinc finger

## Several distinct classes of weak interactions drive the formation of a score of different biomolecular condensates

As described in other articles of this special issue and recent reviews [1], a score of biomolecular condensate is essential for the efficient subcellular organization of the cell. They perform many vital physiological functions, but they are also implicated in cancer [2, 3], neurodegenerative diseases [4, 5] and viral infections [6]. During the last decade of the 20th century, it became clear that sphingolipids and cholesterol undergo two‐dimensional phase separation in the cell membrane to form special domains called ‘lipid rafts’ [7]. These 2D condensates also concentrate proteins modified with a glycosylphosphatidylinositol anchor. The specific localization and concentration of such proteins into lipid rafts play key roles in signalling and vesicle transport.

Several years after these foreshadowing findings, germline granules were discovered. They also have nonaqueous liquid properties and concentrate certain mRNAs and proteins necessary for early embryonic differentiation [8]. This pioneering study stimulated a general reappreciation of three‐dimensional subcellular entities such as germ bodies, Cajal bodies, stress granules and nucleoli, and now, these all have been recognized as being distinct liquid phases [1]. Their most notable features include (a) a content enriched in certain proteins, RNA and metabolites, while excluding some water; (b) rapid formation and dissociation in response to cell conditions; (c) stabilization due to weak and ephemeral interactions such as hydrophobic interactions; (d) molecules within the condensate can exchange from the condensed to the dispersed phases; and (e) fusion with condensates of the same class, but repulsion of other types of condensates. This fifth property is more highly developed in stress granules, which contain two nonmixing layers [9] and nucleoli [10], which consist of three separate layers. The complex organization of the nucleolus was proposed to constitute an assembly line for efficient ribosome biosynthesis [10].

The mutual avoidance of different types of condensates implies that their stabilizing interactions must be more sophisticated than simple hydrophobic interactions [11]. In fact, to date several different kinds of stabilizing contacts have been identified, ranging from π–π and cation–π interactions between small groups of atoms in aromatic and cationic residues [12] to hydrophobic α‐helices [13] to the specific yet weak interactions among entire folded domains such as the N‐terminal domain of transactive response DNA‐binding protein of 43 kDa [14] or the pentamerization domain of nucleophosmin [15]. In some cases, different classes of weak contacts, namely hydrophobic, π‐π and sp^2^‐π interactions, as well as hydrogen bonds, combine to promote condensate formation [16]. Sp^2^ interactions include those formed by delocalized electrons in backbone and side‐chain amide groups and aromatic moieties. In addition to these interactions, there are cases of proline‐rich PPII helices interacting with folded domains to stabilize condensates [1], and even a case of a putative PPII helix formed by nonproline residues interacting with a folded domain to drive condensate formation has been reported [17]. Here, we will review the reported cases of PPII helices contributing to condensate formation and our recent proposal that PPII helices formed by glycine‐rich PPII helices may interact with each other or other partners to promote the formation of condensates [18, 19].

## The unique structure and interactions of polyproline II helices

The PPII helix was first characterized in peptides composed of proline residues in aqueous solution in the 1950s [20]. Compared to the well‐known right‐handed α‐helix, the PPII helix is left‐handed and makes one turn exactly every three residues. Replacing every third Pro residue with Gly allows three PPII helices to associate as tiny glycine can fit and form H‐bonds at the small, interhelical position. This PPII triple helix is the basis of collagen, the most abundant protein in the human body. Other residues besides proline can also adopt the PPII helical conformation. For example, peptides composed of charged residues such as lysine or glutamic acid form PPII helices [21]. More interestingly, segments rich in glycine residues can form bundles of PPII helices as observed in a number of natural proteins, for example acetophenone carboxylase [22]. Many ‘loop’ segments in globular proteins [23] and ‘unfolded’ segments of intrinsically disordered proteins [24, 25] actually adopt the PPII conformation.

An isolated PPII helix is stiff and exposes both side chains and backbone to solvent. These properties favour association with other proteins. For example, in the striking structure of human acetylcholinesterase, the tetramerization motif consists of a central PPII helix bound to four α‐helices [26]. Moreover, PPII helices frequently bind folded domains to mediate protein/protein interactions. Currently, eight classes of PPII helix‐binding proteins/protein domains have been reported: (a) the class II major histocompatibility complex (class II MHC), (b) the glycine–tyrosine– phenylalanine domain, (c) the enabled VASP homology domain, (d) the ubiquitin E2 variant (UEV) domain, (e) the tryptophan–tryptophan (WW) domain, (f) the octamer repeat of aromatic residues domain, (g) the Src‐homology 3 (SH3) domain and (h) profilin. Many of these domains are small, have the N and C termini close together to facilitate modular protein architectures with domain repeats and use exposed aromatic residues to bind a PPII helix. Some of their characteristics are shown in Table 1. In the following paragraphs, we highlight the roles of some of these domains in some particularly fascinating biomolecular condensates.

**Table 1:** Characteristics of PPII‐binding domains.

Domain /Protein	No. of Residues	Biological Role	Binds to	Structure^a^	Reference
Class II MHC	1214	Displays pathogen‐derived peptides for T‐cell activation	Non‐human protein fragments	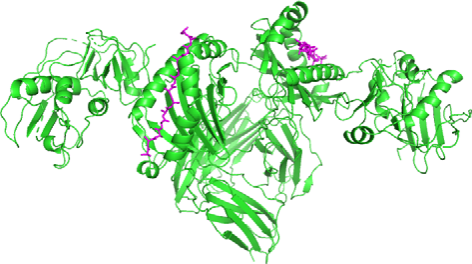	[67, 68]: PDB 1SEB
GYF domain	62	CD2 signaling, immune cell activation	SHRPPPPGHRV	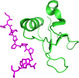	[69, 70]: PDB:1L2Z
EVH domain	115	Actin‐based motile and neural development processes	EFPPPPT	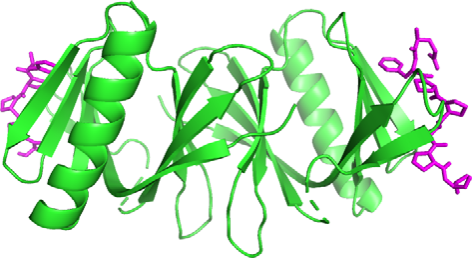	[71]: PDB:1QC6
UEV domain	145	Cytokinesis, viral particle budding	PSAP; PTAP	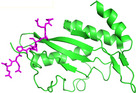	[72]: PDB: 1M4Q
WW domain	35–40	Cytoskeleton, Hippo signaling. Many others	PPxY; LPxY; PPR; PGM motifs; PR; phosphoS‐P; phosphoT‐P	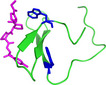	[73]: PDB 1K9Q
OCRE domain	55	Alternative splicing regulation; binds splisosome protein SmN	RPPPPGIR	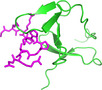	[74, 75]: PDB: 5MF9
SH3 domain	60	Cytoskeleton regulation, condensate formation	R,KxxPxxP; PxxPxR,K	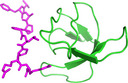	[76]: PDB: 1PRL
Profilin	139	Promotes actin filament formation	S,A,T,G(PPPP..)L	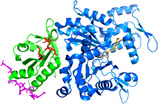	[77, 78]: PDB: 1CJF

^a^ For each structure, the PPII helical ligand is shown in magenta and the binding domain is shown in green. For the WW domain, the two conserved Trp residues are shown in blue. In the case of profilin, residues whose mutation is linked to ALS are shown in red and actin is shown in blue.

## A PPII helix at the C terminus of RNA polymerase mediates the formation of both superenhancer condensates and nuclear paraspeckle condensates for splicing

One triumph of structural biology was the elucidation of the conformation and mechanism of RNA polymerase II (RNA pol II) and transcription factors [27], for which Roger Kornberg received the Nobel Prize in 2006. These discoveries currently aid the understanding of viral RNA polymerases, such as the SARS‐CoV‐2 replicase. However, one domain of RNA pol II initially eluded elucidation: over 350 residues at the C terminus were missing in the X‐ray structure. The sequence of this absent C‐terminal domain (CTD) consists of 52 repeats of a consensus seven‐residue sequence: YSPTSPS. Robert Woody, a top expert in circular dichroism, used this spectroscopic technique to reveal that the conformational ensemble of RNA pol II CTD is mainly statistical coil with a significant proportion of PPII helix [28]. He and his team predicted that transcription factors with WW or SH3 domains could use this PPII helix to bind RNA pol II.

One such protein is Mediator, which is in fact a large set of protein factors that interact with RNA pol II and DNA regulator sequences distant from the transcribed gene to strongly enhance or repress transcription [29]. When bound to Mediator, the CTD of RNA pol II adopts two PPII helices connected by a turn [30]. Work over the last 15 years has shown that the Mediator complex is essential for forming a phase‐separated ‘superenhancer’ condensate, which dramatically increases transcription rates and plays important physiological roles as well as in cancer [31].

Despite the elegance and importance of this mechanism, Sharp and others wondered whether the RNA Pol II CTD could be moonlighting. They realized that the CTD heptad repeat is rich in Ser, Tyr and Thr residues, which can be phosphorylated, and might endow the RNA Pol II CTD with the ability to interact with a second set of partners. In 2019, they reported that following phosphorylation by CDK7/CDK9, the RNA Pol II CTD stops interacting with Mediator and the enhancer complex and instead nucleates the formation of a second type of biocondensate, called nuclear paraspeckles, by binding to arginine‐rich splicing factors of the spliceosome [32]. This phosphorylated form of RNA Pol II also adopts a PPII helix [33] or a mixed PPII/extended conformation [34] when bound to partners. These findings are reminiscent of an early scene from the film ‘Cinema Paradiso’ (https://www.youtube.com/watch?v=qMqE1Fayk28) where the village priest signals Alfredo, the cinema technician, to mark the kissing scenes for cutting and splicing. In an analogous fashion, the phosphorylation of RNA pol II CTD (the priest’s bell) disrupts the association of the enhancer complex (kissing couples) and induces the formation of the spliceosome/nuclear paraspeckles (Alfredo and his scissors).

## Two‐faced profilin binds actin and PPII helices to modulate condensate formation

Profilin is a small protein with separate binding sites for actin and PPII helices. It is well known for promoting changes in the actin cytoskeleton for cell development and motion. First, profilin binds to an actin monomer and promotes ADP→ATP exchange. Then, profilin–actin pairs are channelled into growing actin filaments as profilin binds to PPII helices in proteins such as WASP and VASP. In Huntington’s disease, toxic monomers and aggregates of the N‐terminal fragment of the huntingtin protein (Htt NTF) disrupt normal phase separation processes and induce necrosis and apoptosis [35]. These Htt NTFs contain an expanded polyQ tract followed by polyproline segments, which adopt polyproline II helices with occasional kinks [36]. Interestingly enough, profilin can bind to the PPII tracts of Htt NTF and reduce their cytotoxicity [37, 38].

Profilin is also essential for the formation of an extensive actin filament network at dendritic spines [39], which is essential for memory consolidation [40]. This actin filament network is highly dynamic, turning over within minutes [41], so how can some memories last a lifetime? Cytoplasmic polyadenylation element‐binding protein (CPEB) whose functional aggregation is essential for long‐term memory [42] is present in P‐body‐like neuronal granules at dendritic spines [43]. CPEB’s folded RNA recognition motif and ZnF domains retain and repress mRNAs, which are key for memory consolidation. Following stimulation, CPEB’s N‐terminal disordered region, which contains polyglutamine and polyproline segments, forms a highly stable functional amyloid [44] leading to the release and activation of the retained mRNAs. As a working hypothesis, we recently proposed that after amyloid formation, the PPII helices by human CPEB3’s polyproline tracts could be favourably positioned to interact with profilin and orchestrate a permanent fortification of the local actin cytoskeleton [45].

## SH3 domains reveal how affinity and avidity control aqueous ↔ liquid condensate ↔ solid‐phase transitions

**Fig. 1:**
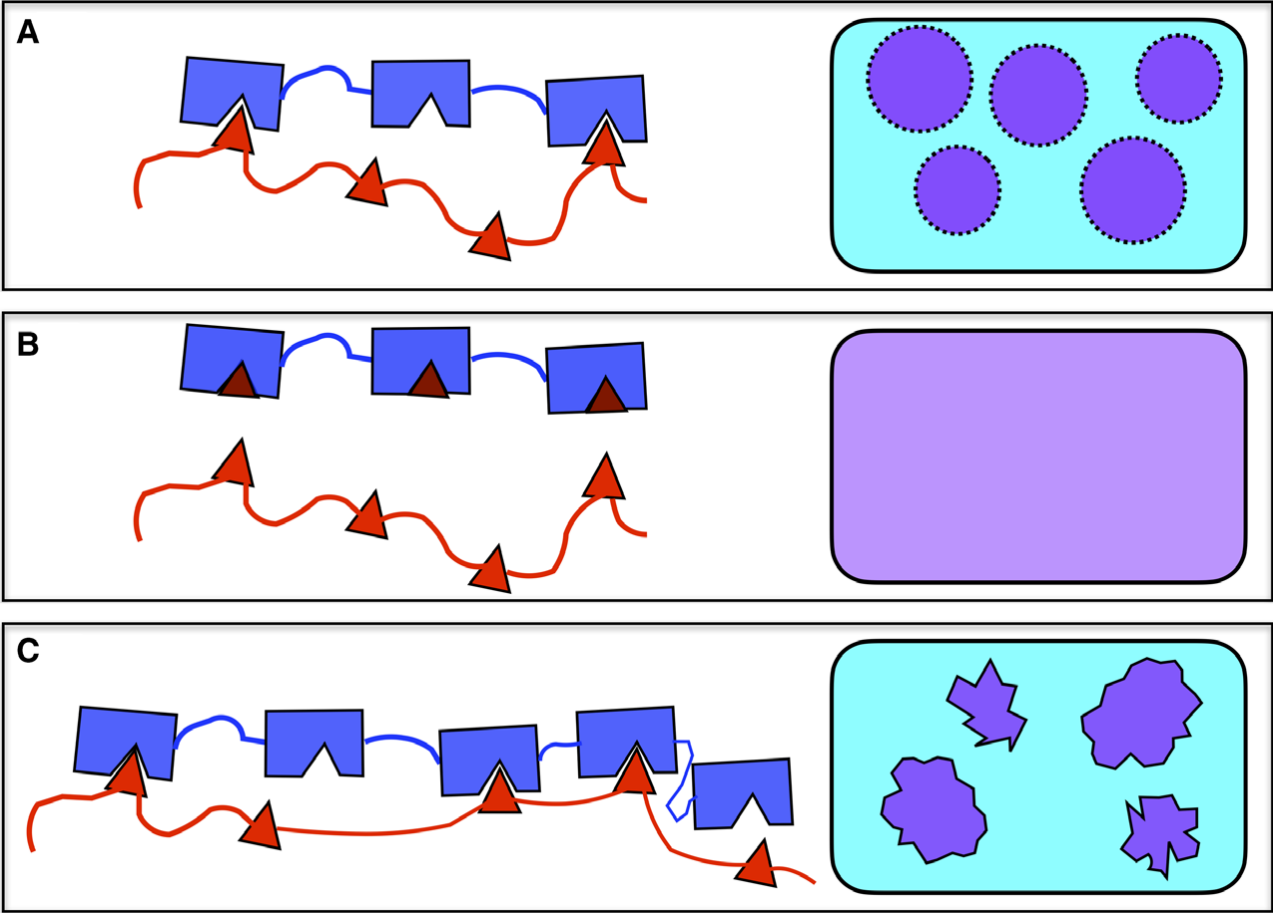
Formation of soluble oligomers, phase‐separated liquid microdroplets and hydrogels by SH3 domains + PPII helices. (A) Three modular SH3 domains (blue) with moderate affinity for four PPII helices linked on the same polypeptide chain (red) form phase‐separated liquid microdroplets (right, purple). (B) The addition of high‐affinity PPII helical monomers (dark red) leads to the displacement of the moderate‐affinity ligands (red) from the SH3 domains (blue) and the dissolution of the microdroplets. (C) Increasing the number of linked SH3 domains (blue) and moderate‐affinity PPII helices (red) to five each leads to the formation of rigid hydrogels (right, purple). Figure based on the results of Ref. [47].

The SH3 domain is the most common and versatile PPII helix‐binding element [46]. Like most PPII‐binding motifs, the SH3 domain is modular in nature, as many proteins have a few or several SH3 domains strung close together along their sequence. For example, the protein Nck contains three SH3 domains and binds to six proline‐rich motifs (PRMs), which putatively adopt PPII helices, in a second protein called N‐WASP to form part of the glomerular filtration barrier in kidney podocytes. In 2012, Li *et al*. [47] exploited this system to uncover how changing the strength of the SH3 domain/PRM interaction, as well as the number of interacting SH3 domains and PRM, affects phase transitions. They discovered that when a single SH3 domain binds to a high‐affinity PRM, a stable heterodimer is formed, which remains in the aqueous phase (Fig. 1A). In contrast, when an aqueous solution containing polypeptides with three SH3 domains (SH3)_3_ with moderate affinity for PRM is mixed with a second solution containing polypeptides with four PRM (PRM)_4_, liquid/liquid phase separation occurs as the polypeptides combine to form condensates (Fig. 1B). It is fascinating that the addition of a monovalent, high‐affinity PRM to this system causes the condensate to break up, as the tight binding ligand displaces the low‐affinity, medium‐avidity (PRM)_4_ polypeptide. As the reader might have already guessed, moderate‐affinity systems with more SH3 domains and PRMs, such as (SH3)_5_ plus (PRM)_5_, produced semi‐solid, gel‐like condensates (Fig. 1C) [47].

## Recognition of noncanonical segments by an SH3 domain can also modulate condensation formation

Whereas most SH3 domain ligands contain proline residues, SH3 domains that bind class I ligands can also recognize an RKXXYXXY motif, where X is a small, nonproline residue [48]. As such proline‐less segments are common, their interactions with SH3 domains could impact condensate formation. Certain mRNAs are transported along dendrites in a type of condensate called ‘neuronal granules’ by the protein hnRNPA2. The mRNA cargo is released when the kinase Fyn is incorporated into the neuronal granule at its destination and phosphorylates the hnRNPA2 [49]. Fyn uses its SH3 domain to bind to hnRNPA2. However, hnRNPA2 does not contain the PXXP motif recognized by SH3 domains. Intrigued by this observation, Amaya, Ryan and Fawzi recently used NMR spectroscopy to study the interaction between the C‐terminal disordered region of hnRNPA2 with Fyn [17]. They found evidence that Fyn may bind to residues Y_335_GGRSRY_341_, in the ‘disordered’ region of hnRNPA2, right at the C terminus of hnRNPA2. Interestingly, if this segment were to adopt a PPII helix, the R and Y side chains would be favourably positioned to form cation–π interactions. This suggests that segments lacking PRMs may be able to adopt the PPII helical conformation and bind to SH3 domains to contribute to biomolecular condensate formation and dissociation.

## Proteins formed by bundles of Gly‐rich PPII helices have diverse biological functions

**Fig. 2:**
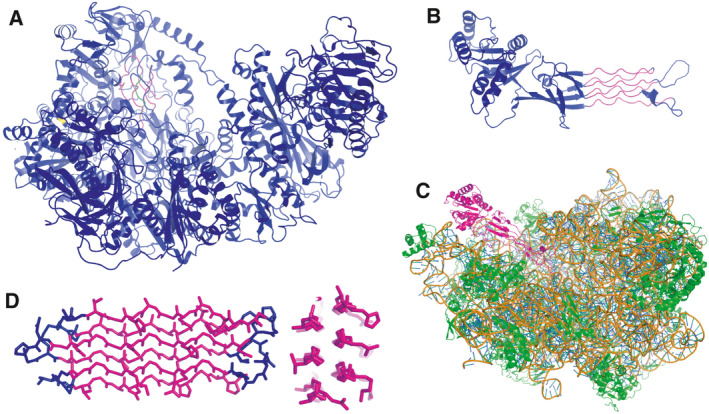
Glycine‐rich PPII helical bundles are found in a variety of proteins. (A) Ribbon diagram of the *Aromatoleum aromaticum* acetophenone carboxylase core complex (PDB 5L9W). α‐helices, β‐strands and loops are shown in blue, and PPII helices are shown in magenta except for the central PPII helix, which is shown in green. (B) X‐ray crystal structure of the *Bacillus subtilis* Obg GTPase (PDB: 1LNZ) with α‐helices, β‐strands and loops are shown in blue, and PPII helices are shown in magenta. (C) Cryo‐EM structure of the *E. coli* Obg GTPase bound to the large ribosomal subunit, where it acts to mimic a tRNA and block ribosome assembly (PDB: 1LN7). Here, the entire Obg GTPase is shown in magenta, ribosomal proteins are shown in green and rRNA is shown in gold (backbone) and blue (bases) (PDB: 4CSU). (D) The *Hypogastrum* ‘snow flea’ antifreeze protein contains six PPII helices (magenta) connected by short loops (blue). The cross section (right) shows the polar (right side) and nonpolar (left) faces (PDB: 3BOG).

Whereas the polyproline II helix is commonly associated with proline residues, it can also be adopted by proteins rich in glycine. These proteins share a conformation based on that of polyglycine peptides, which adopt a network of PPII helices stabilized by interhelical N‐H|||O=C [50] and Cα‐H|||O=C hydrogen bonds [51]. In the case of acetophenone carboxylase, the β‐subunit contains a complete hexagonal bundle with six PPII helices surrounding a central seventh PPII helix [22] (PDB 5L9W; Fig. 2A). This PPII domain is mostly buried inside this enzyme. Based on this crystal structure and sequence analysis, [52] Schühle and Heider proposed that this fold would be shared by all bacterial enzymes of the hydantoinase/carboxylase family.

The GTP‐binding protein Obg, a completely unrelated protein, also contains a glycine‐rich PPII helical bundle, but in this case, the crystal structure showed that the bundle is mainly solvent‐exposed and consists of six PPII helices arranged in two layers (PDB 1LNZ; Fig. 2B) [53]. This essential domain is conserved from bacteria to humans, and remarkably, it acts as a tRNA mimic that binds the large ribosomal subunit to regulate ribosomal assembly (PDB 4CSU; Fig. 2C) [54].

A remarkable domain consisting of ten PPII helices is found at the tip of the spike protein of T‐even (except T4) bacteriophages [55]. These helices are arranged in three layers, with the two central PPII helices being composed of six and seven consecutive glycine residues, similar to the structure of polyglycine [50]. Bacteriophages are extremely abundant; for every grain of sand on Earth, there are about a trillion phages [56]. This means that this glycine‐rich helical bundle fold is exceptionally plentiful in the biosphere. Whereas glycine‐rich PPII bundle proteins are rare relative to those composed by α‐helices and β‐sheets, their abundance, presence across different biological kingdoms and diversity of folds, which likely evolved independently, evince the success of this protein structure family.

Glycine‐rich PPII helical bundles are also observed in the *Collembola Hypogastruridae* ‘snow flea’ antifreeze proteins [57]. Initially isolated in Canada, similar *Collembola* proteins have been recently isolated in the Middle East [58], Iceland [59] and Japan [60]. As in Obg, their structures consist of a flat, solvent‐exposed, two‐layer network of PPII helices (PDB 3BOG; Fig. 2D). However, the snow flea antifreeze proteins can have between six and 13 PPII helices. One face is rich in exposed Ala residues, poor in charged residues and has been reported to bind to nascent ice crystals [61]. A similar fold has also been proposed to be present in a high glycine–tyrosine hair keratin‐associated protein [62]. Based on sequence comparison, CD and FTIR spectroscopy results and MD simulations, these researchers advanced a structural model featuring four glycine‐rich PPII helices stabilized by two disulfide bonds.

In summary, proteins or protein domains composed of Gly‐rich PPII helical bundles have been found in diverse proteins across the kingdoms of life. Both the widespread nature of this fold and the observation that it is overlooked by the most popular secondary structure classification algorithms [22, 23] lead us to suggest that it may also be present yet unrecognized in other biological contexts.

## Could Gly‐rich PPII helices contribute to condensate formation?

The several examples reviewed here highlight how PPII helices can interact with folded globular domains to stabilize biomolecular condensates. Is it possible that condensate formation could also be promoted by Gly‐rich PPII helices associating with each other? Condensate formation by fused in sarcoma (FUS) protein is driven by cation–π interactions between its RGG motifs and G/S,Y,G/S repeats [12]. The abundance of glycine and the spacing of Arg residues in the former match the template seen in folded PPII helical bundle proteins such as ‘snow flea’ antifreeze protein (sfAFP) (Fig. 2D, [57]), so RGG motifs or similar (RGGF)_N_ repeats in the CTD of nucleolin might form PPII helices. While there are well‐developed tools based on NMR chemical shift deviations to identify α‐helices and β‐sheets and to measure their population in proteins, none were available for PPII helices.

Spurred by this need, we recently characterized ^13^C,^15^N‐labelled *Hypogastrura harveyi* sfAFP by NMR spectroscopy [18]. This 81‐residue protein is composed of 46% glycine and 10% alanine residues, yet it adopts a well‐ordered, brick‐shaped structure consisting of six long PPII helices [61]. We found that PPII helical bundles have a signature set of ^13^Cα, ^13^Cβ, ^13^CO and ^1^Hα chemical shift deviations [18]. Most remarkably, when a glycine residue is at an internal position, one of its two ^1^Hα nuclei shows a highly anomalous chemical shift value. The formation of weak Cα‐H|||O=C hydrogen bonds by these ^1^H nuclei can account for this extraordinary value.

Through H/D exchange and {^1^H}‐^15^N dynamic measurements, we found that sfAFP has a conformational stability and rigidity similar to those of global proteins composed of α‐helices and β‐sheets. This is perplexing if we consider that glycine residues enjoy a high conformational entropy in the unfolded state, and like hippies called to join a military formation, are loath to give up their freedom and become fixed in a folded protein [63]. Stabilizing contributions from disulfide bonds, backbone N‐H|||O=C and Cα‐H|||O=C hydrogen bonds and hydrophobic interactions at a dimer interface [18, 64], as well as n→π* interactions [65], are key to overcoming this entropy effect. These findings should facilitate the use of PPII helical bundles as a new structural element for protein design, which has made remarkable achievements in recent years despite being limited to α‐helices and β‐sheets [66]. Finally, whereas the form of sfAFP with six PPII helices is the most abundant, natural samples also contain a longer isoform composed of 13 PPII helices with a similar, though longer disposition [51, 58]. This suggests that two PPII helical bundles composed of several PPII helices may combine at the ends to form a larger PPII helical bundle. On a speculative note, such a (PPII)_6_ + (PPII)_6_ association event might also contribute to condensate formation.

## Conclusions

Biomolecular condensates play essential roles in multiple physiological functions such as ribosome synthesis, transcription, splicing, stress response, RNA transport and translation regulation. On the other hand, they are also implicated in cancer, neurodegenerative diseases and viral infections. Biomolecular condensate formation and dissociation are governed by several classes of weak, transient interactions, one of which could well be the weak binding of small domains to PPII helices. Since recent work has shown that PPII helices and helical bundles can be formed by polypeptides poor in proline, their role in condensate formation may well be larger than what has previously been recognized.

## Conflict of interest

The authors declare no conflict of interest.

## Author contributions

MM, JO and DVL researched the literature and wrote the paper.

## Data availability

There are no related papers.
